# Altered splicing of CEACAM1 in breast cancer: Identification of regulatory sequences that control splicing of CEACAM1 into long or short cytoplasmic domain isoforms

**DOI:** 10.1186/1476-4598-7-46

**Published:** 2008-05-28

**Authors:** Shikha Gaur, John E Shively, Yun Yen, Rajesh K Gaur

**Affiliations:** 1 Division of Molecular Biology, Beckman Research Institute of the City of Hope, Duarte, CA 91010, USA; 2 Department of Clinical & Molecular Pharmacology, Beckman Research Institute of the City of Hope, Duarte, CA 91010, USA; 3 Division of Immunology, Beckman Research Institute of the City of Hope, Duarte, CA 91010, USA; 4 Graduate School of Biological Sciences, Beckman Research Institute of the City of Hope, Duarte, CA 91010, USA

## Abstract

**Background:**

Carcinoembryonic antigen-related cell adhesion molecule 1 (CEACAM1), a cell adhesion molecule expressed in a variety of cell types is a putative tumor suppressor gene. Alternative splicing of CEACAM1 generates 11 different splice variants, which include 1–4 ectodomains with either short or long cytoplasmic domain generated by the exclusion (CEACAM1-S) or inclusion (CEACAM1-L) of exon 7. Studies in rodents indicate that optimal ratios of CEACAM1 splice variants are required to inhibit colonic tumor cell growth.

**Results:**

We show that CEACAM1 is expressed in a tissue specific manner with significant differences in the ratios of its short (CEACAM1-S) and long (CEACAM1-L) cytoplasmic domain splice variants. Importantly, we find dramatic differences between the ratios of S:L isoforms in normal breast tissues versus breast cancer specimens, suggesting that altered splicing of CEACAM1 may play an important role in tumorogenesis. Furthermore, we have identified two regulatory *cis*-acting elements required for the alternative splicing of CEACAM1. Replacement of these regulatory elements by human β-globin exon sequences resulted in exon 7-skipped mRNA as the predominant product. Interestingly, while insertion of exon 7 in a β-globin reporter gene resulted in its skipping, exon 7 along with the flanking intron sequences recapitulated the alternative splicing of CEACAM1.

**Conclusion:**

Our results indicate that a network of regulatory elements control the alternative splicing of CEACAM1. These findings may have important implications in therapeutic modalities of CEACAM1 linked human diseases.

## Background

Alternative splicing is a process by which a single pre-mRNA can generate multiple mRNA isoforms by differential joining of 5' and 3' splice sites (ss) [1,2]. Mapping of the human genome suggests that more than 70% of genes encode for transcripts that undergo alternative splicing [3-5], and most alternative splicing events affect coding capacity of genes [6,7]. Given the importance of mRNA splicing in gene expression, it is not surprising that ~50% of human genetic diseases may be caused by mutations of the splice junctions or auxiliary regulatory sequences [8,9]. However, sequencing of many cancer specific alternatively spliced genes has indicated that genomic mutations are not the only cause of aberrant splicing. In fact, deregulated expression of splicing factors has been shown to affect alternative splicing, which is often accompanied by a shift in the ratio of alternatively spliced mRNA isoforms [10-15]. Accordingly, molecular strategies that modulate alternative splicing have been the focus of attention over last several years [16-20].

**Figure 1:**
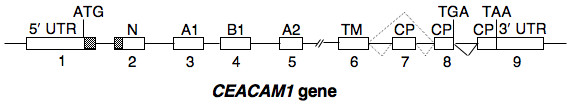
**Schematic representation of human *CEACAM1 *gene and its alternative splicing into long and short cytoplasmic domain isoforms.** Open boxes or lines represent exons and introns, respectively. Shaded portions of exons denote the leader sequence. The broken lines represent inclusion or exclusion of exon 7 thereby generating CEACAM1-L or CEACAM1-S splice variants, respectively. Numbering of the exons is indicated below each exon. N, A1, B1 and A2 refer to the Ig domains [54]. UTR, untranslated region; ATG, the start codon; TM, transmembrane domain; CP, cytoplasmic domain; TGA and TAA, the stop codons for short and long cytoplasmic domain isoform, respectively.

CEACAM1 (carcinoembryonic antigen-related cell adhesion molecule 1), also known as biliary glycoprotein [21] or CD66a [22], is a transmembrane protein of the immunoglobulin superfamily [23]. It consists of N-terminal ectodomain, either two or four extracellular Ig-like domains, a conserved transmembrane domain, and either a short (12–14 amino acids, CEACAM1-S) or long cytoplasmic domain (72–74 amino acids, CEACAM1-L) generated due to the alternative splicing of CEACAM1 pre-mRNA. The inclusion of exon 7 in CEACAM1-L mRNA shifts the open reading frame resulting in the use of a distant stop codon and expression of a long cytoplasmic domain (72–74 amino acids). On the other hand, exclusion of exon 7 produces a proximal stop codon thereby generating a shorter mRNA that translates into a short cytoplasmic domain (12–14 amino acids) [24,25] (Fig. 1). The long cytoplasmic domain encodes two ITIMs (immunoreceptor tyrosine-based inhibitory motif) that bind SHP-1 when phosphorylated and convey inhibitory activities to CEACAM1-L [26]. The short cytoplasmic domain has been shown to bind actin, tropomyosin, calmodulin, and annexin II [27,28] and is involved in lumen formation [29].

CEACAM1 is expressed in a polarized luminal orientation in the liver, colon and breast. It is downregulated in various types of tumors such as colorectal carcinomas, hepatomas, breast carcinomas, and prostate carcinomas [30-33], suggesting a tumor suppressive role for CEACAM1. Indeed, CEACAM1-L has been shown to possess tumor growth inhibitory activity when its gene is transfected and expressed in prostate [34], bladder [35] or breast cancer cells [36]. In addition, CEACAM1-L decreases cell growth in response to insulin [37,38]. However, tumor suppressive role of CEACAM1-L has been questioned by other findings. For example, the expression of CEACAM1-L is upregulated in primary lung carcinomas when compared with adjacent normal lung tissue [39]. Similarly, CEACAM1-L is expressed abundantly in several human colon and thyroid carcinoma cell lines (this study and see ref [25,40]). Importantly, in contrast to previously held notion that the long cytoplasmic domain isoform of CEACAM1 possesses tumor suppressive properties, we recently showed that forced expression of CEACAM1-S revert the malignant mammary epithelial cell line MCF7, which expresses low levels of CEACAM1-L, to a normal acinar morphology [29]. Together, these studies raise the possibility that an optimal ratio of CEACAM1 S:L splice variants may be important for maintaining normal cell function. This is consistent with a study in rodents where optimal ratios of CEACAM1 cytoplasmic domain splice variants have been found to be essential for inhibiting colonic tumor cell growth [41].

In this report, we set out to answer three important questions: 1) Do CEACAM1 transcripts undergo alternative splicing in a tissue specific manner? 2) Is splicing of CEACAM1 altered in breast cancer? and 3) What are the *cis*-acting element(s) in exon 7 that regulate CEACAM1 alternative splicing in two different cytoplasmic domain isoforms? Here we show that the ratios of CEACAM1 splice variants vary significantly among a variety of normal human tissues and cell lines derived from human tumors. We further demonstrate that compared to normal tissues, the ratios of CEACAM1 short to long cytoplasmic domain splice variants vary significantly in breast cancer specimens. Finally, we report the identification of two regulatory *cis*-acting elements that control the alternative splicing of CEACAM1.

## Results

### CEACAM1 long and short cytoplasmic domain splice variants are expressed differentially in normal human tissues

Alternative splicing of CEACAM1 generates two cytoplasmic domain splice variants that differ by the inclusion or exclusion of exon 7 (Fig. 1). As a first step towards understanding the relationship between altered splicing of CEACAM1 transcripts and neoplasia, we decided to examine the expression of CEACAM1 long and short cytoplasmic domain splice variants in a variety of human tissues. Total RNAs from normal human tissues (brain, colon, heart, kidney, liver, lung and prostate) were subjected to RT-PCR with primers that were designed to specifically co-amplify CEACAM1-L (408-bp) and CEACAM1-S (355-bp) mRNA splice variants.

**Figure 2:**
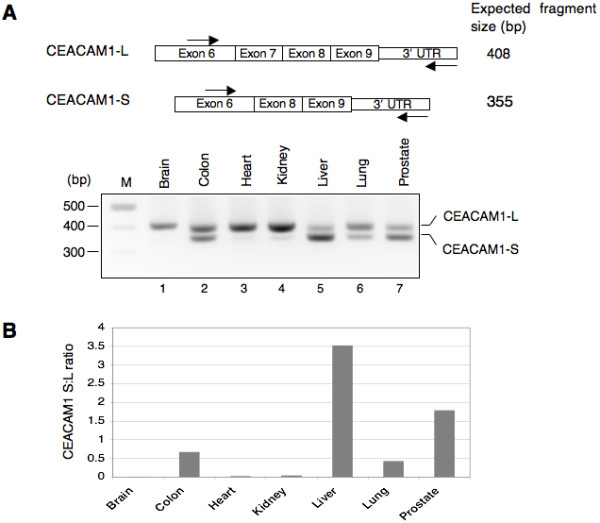
**CEACAM1 is differentially expressed in normal human tissues.** (A) Schematic diagram of RT-PCR strategy for the identification of CEACAM1 long and short cytoplasmic domain splice variants. Binding sites for the forward and reverse primers and the expected size of amplified fragments are shown. The PCR amplified products were separated by electrophoresis on a 2.5% agarose gel. M, refers to 100 bp DNA ladder (NEB). (B) Histogram shows CEACAM1-S/CEACAM1-L (S:L) ratio quantified with NIH ImageJ program.

Fig. 2A demonstrates that colon, liver, lung and prostate co-expressed CEACAM1-L and CEACAM1-S splice variants. While liver and prostate expressed higher levels of CEACAM1-S splice variant, colon and lung expressed more of the CEACAM1-L splice variant (Fig. 2A, compare lanes 2, 5, 6 and 7). In contrast, brain, heart and kidney almost exclusively expressed the CEACAM1-L splice variant (Fig. 2A, cf. lanes 1, 3 and 4). Even more striking are the differences between the ratios of CEACAM1-S and CEACAM1-L splice variants. For example, although colon, liver, lung and prostate expressed both cytoplasmic domain isoforms of CEACAM1, the S:L ratio in liver is significantly higher than other tissues. Quantitation of the data shown in Fig. 2B indicate that the ratio of S:L splice variants in liver is 2, 5 and 8-fold higher than in prostate, colon and lung, respectively.

### Splicing of CEACAM1 exon 7 is altered in cancer cell lines

To determine whether altered splicing of CEACAM1 is linked to tumorigenesis, we examined the splicing profile of CEACAM1 transcripts in a variety of human cancer cell lines derived from brain, breast, colon and prostate. The rationale for choosing these cell lines was based on the fact that CEACAM1 has been extensively studied in colon [42,43], prostate [34,44] and breast carcinomas [45]. Therefore, cell lines derived from these tissues should serve as model system for assessing whether altered CEACAM1 splicing is associated with cancer. Since very little is known about the role of CEACAM1 in brain cancer, we also examined the expression of CEACAM1 in three human malignant glioma cell lines, U87MG, U251T and T98G. Glioma is a rapidly fatal cancer in which the search for oncogenes and tumor suppressor genes is important.

**Figure 3:**
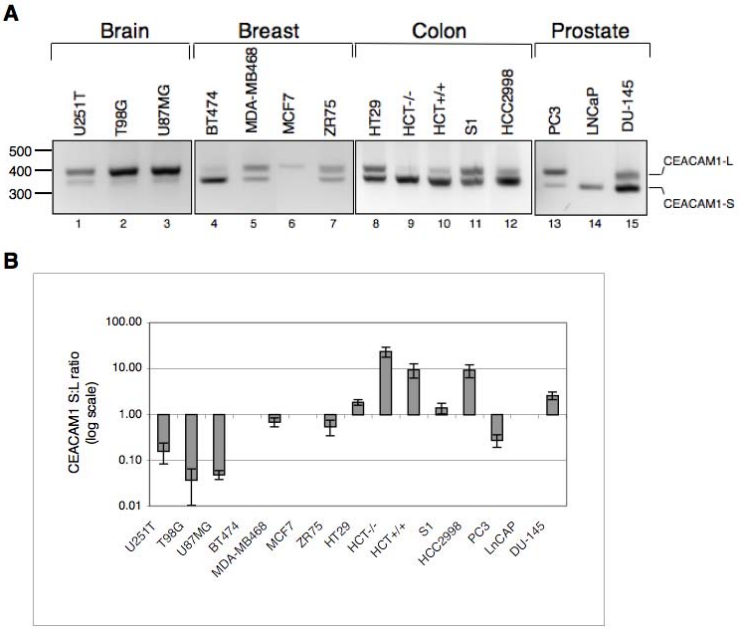
**The expression profile of CEACAM1 in human cancer cell lines. **(A) Total RNAs from the indicated cell lines were isolated and subjected to RT-PCR as described in Figure 2. The position of CEACAM1 long and short cytoplasmic domain splice variants is indicated on right. (B) Histogram depicting CEACAM1-S/CEACAM1-L (S:L) ratio in a logarithmic bar graph quantified with NIH ImageJ program. Data represent the mean ± SD of at least three independent experiments.

The results of the RT-PCR assay shown in Fig. 3A demonstrate marked differences in the expression of CEACAM1 long and short cytoplasmic domain splice variants. Human malignant glioma cell lines T98G and U87MG mainly expressed the CEACAM1-L splice variant. Although U251T cells expressed relatively lower levels of CEACAM1-L, the S:L ratio in these cells (0.24) is significantly higher compared to T98G (0.06) and U87MG (0.05) cells (Fig. 3A cf. lanes 1–3 and see Fig. 3B). Colon cancer cell lines, with the exception of HT29 and S1, expressed for the most part short cytoplasmic domain splice variant (Fig. 3A cf. lanes 8–12). The results with breast and prostate cancer cell lines were somewhat mixed. While breast cancer cell lines MDA-MB468 and ZR75 expressed both splice variants, BT474 cells expressed only the short splice variant (Fig. 3A cf. lanes 4–7). The observation that MCF7 cells barely expressed CEACAM1 is consistent with our earlier study [29]. Among the prostate cancer cell lines examined, PC3 and DU-145 expressed both the short and long cytoplasmic domain splice variants of CEACAM1: DU-145 cells expressed more of the short splice variant, PC3 cells largely expressed the long splice variant, and LNCaP cells expressed only the short splice variant (Fig. 3A cf. lanes 13–15).

A comparative analysis of S:L ratios between normal human tissue and corresponding cancer cell line revealed further insights. For example, normal colon co-expressed both splice variants with S:L ratio of 0.67 (Fig. 2B). In contrast, colon cancer cell lines expressed elevated levels of short splice variant with S:L ratios ranging between ~1.6–21 (Fig. 3). The observed S:L splice variant ratio in PC3 (0.2) and DU-145 (2.3) cells was different compared to normal prostate (1.7). Since LNCaP cells expressed only the short splice variant, a direct comparison of the S:L ratio between normal prostate and this cell line was not possible. Unlike normal brain tissue that exhibited only CEACAM1-L splice variant, malignant glioma cell lines expressed both splice variants with significant differences between S:L ratios (compare lane 1 in Fig. 2A with lanes 1–3 in Fig. 3A). These data indicate that S:L splice variant ratio in U251T cells is 3.6 and 4.1-fold higher than T98G and U87MG cells, respectively (Fig. 3B).

### Altered splicing of CEACAM1 exon 7 and breast cancer

The results presented in the previous section suggest that CEACAM1 is differentially expressed in various human tissues and apparently alteration in the splicing pattern of CEACAM1 is linked to tumorigenesis. To extend this study further and to ascertain that altered splicing of CEACAM1 mRNA is linked to tumorigenesis, we isolated total RNAs from a number of breast cancer tissues that were deposited in the City of Hope tissue bank. These tissues were surgically removed from patients who were between the ages of 31–64 and had not undergone chemotherapy or radiation treatment prior to surgery.

**Figure 4:**
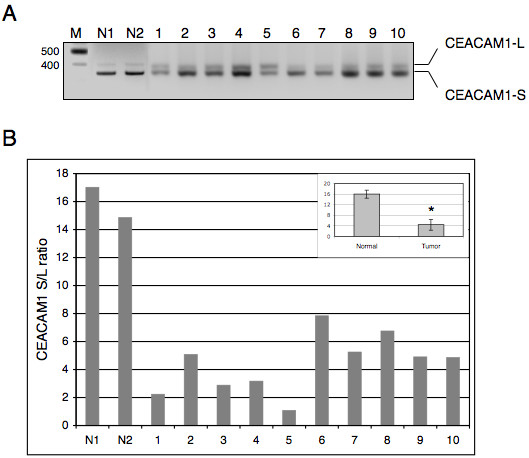
**CEACAM1 pre-mRNA splicing is altered in breast cancer.** (A) CEACAM1 splicing pattern in ten breast cancer specimens (1–10) and two normal-appearing (N1 and N2) breast tissues is shown. See Figure 2 and experimental section for details. (B) Histogram representing the quantification of CEACAM1S/L ratio as determined in Figure 2. *P < 0.007 versus normal.

As shown in Fig. 4, CEACAM1 mRNA splice variants in which exon 7 was excluded accounted for most of the spliced transcripts in normal tissues (see lanes N1 and N2). Quantitation of these data demonstrate that the ratios of S:L cytoplasmic domain splice variants range between ~15–17. In contrast, breast cancer specimens displayed expression of both short and the long cytoplasmic domain splice variants of CEACAM1 with a marked decrease in the ratio of S:L. Unlike normal tissues, the S:L splice variant ratios in breast cancer specimens varied from 1.0 to 7.8 (Fig. 4B). The combined results from normal human tissues, cancer cell lines and breast cancer specimens suggest that alteration of CEACAM1 splicing could be a common feature of breast cancer and an optimal ratio between the short and the long cytoplasmic domain splice variants might be required for normal tissue function.

### Identification of *cis*-acting elements that regulate the alternative splicing of exon 7

**Figure 5:**
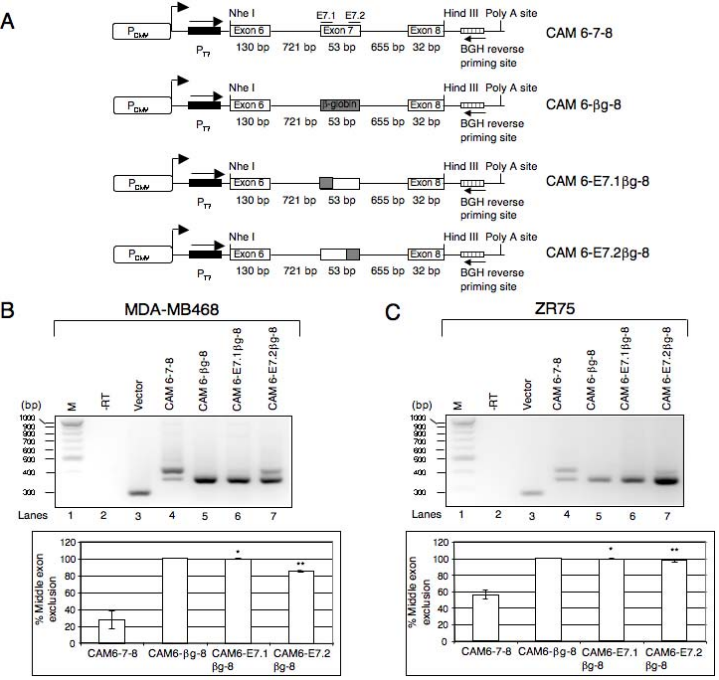
**Identification of CEACAM1 regulatory sequences required for the splicing of exon 7.** (A) Schematic representation of CEACAM1 minigene or chimeras cloned into pcDNA 3.1/Myc-His(-) C. Open or shaded boxes are exons and lines represent introns. The name of each minigene is indicated on right. The size of middle exon in all minigenes is identical to exon 7 of CEACAM1. In CAM 6-βg-8 the entire middle exon is replaced by 53 bp of •-globing exon 2. In minigenes CAM 6-E7.1•g-8 and CAM 6-E7.2•g-8, the first and the last 20 bp (E7.1 and E7.2 regions) of CEACAM1 exon 7 are replaced by •-globin exon 2 sequences. The forward (T7) and reverse priming sites (BGH) are indicated. (B and C) RT-PCR analysis of RNA derived from MDA-MB468 and ZR75 cells transiently transfected with indicated minigenes or empty vector. -RT represents omission of reverse transcriptase during cDNA synthesis. The data shown is representative of three independent experiments. Bar diagrams represent the mean ± SD of at least three independent experiments. *P < 0.001 and **P < 0.01 versus CAM 6-7-8.

It is now widely accepted that regulation of alternative splicing involves multiple *cis*-acting regulatory elements and *trans*-acting splicing regulators. An established approach to identify such regulatory elements is to design transfectable minigenes that could faithfully reproduce a given splicing pattern in a cell line. To test whether a CEACAM1 minigene could recapitulate exon 7 alternative splicing, we PCR amplified a CEACAM1 cassette comprising exons 6, 7 and 8, along with the entire intervening intronic sequences from human genomic DNA. The PCR amplified cassette was cloned downstream of a CMV promoter in a mammalian expression vector yielding a minigene construct (hereafter referred to as CAM 6-7-8, see Fig. 5A).

The breast cancer cell lines MDA-MB468 and ZR75 in which CEACAM1 pre-mRNA undergoes alternative splicing giving rise to both short and long cytoplasmic domain splice variants (see Fig. 3) were transfected transiently with CAM 6-7-8. After 24 h, total RNAs were extracted and the relative abundances of CEACAM1-S and L splice variant mRNAs were estimated by RT-PCR with primers that were designed to amplify specifically the transcripts derived from minigene. We found that CAM 6-7-8 minigene mimics the splicing profile of endogenous CEACAM1 transcripts in both cell lines, albeit with some differences in the ratios of short and long splice variants (compare lane 4 in Fig. 5B and 5C with lanes 5 and 7 in Fig. 3A). To excluded possibility that the observed splicing patterns could be CMV promoter-dependent, we subcloned CAM 6-7-8 minigene downstream of β-actin promoter. Transient transfection followed by RT-PCR assay indicated that although β-actin promoter exhibited lower levels of CEACAM1 transcripts, the splicing profile and the ratios of S:L splice variants were similar to the endogenous transcripts (data not shown). These results demonstrate that the CAM 6-7-8 minigene can be used to identify *cis*-acting sequences that are responsible for controlling the splicing of exon 7.

As a first step to identify the regulatory elements that control exon 7 splicing, we constructed a series of chimeric minigenes and examined the splicing pattern of the transcripts derived from these minigenes in MDA-MB468 and ZR75 breast cancer cell lines. We replaced exon 7 (53 nt) with the first 53 nt of human β-globin exon 2 to yield pCAM 6-βg-8 (Fig. 5A). Splicing profile of CAM 6-βg-8 transcripts in MDA-MB468 and ZR75 cell lines gave rise to spliced mRNAs lacking middle exon, suggesting that exon 7 contains regulatory element(s) that are required for its splicing (see lane 5, Fig. 5B and 5C).

To dissect the exon 7 sequences that contribute to its splicing, we constructed two minigenes in which first (E7.1 region) or the last 20 nucleotides (E7.2 region) were replaced by sequences of human β-globin exon 2 to yield CAM 6-E7.1βg-8 and CAM 6-E7.2βg-8, respectively (Fig. 5A). The transcripts encoded by CAM 6-E7.1βg-8 minigene resulted in skipping of exon 7 in both cell lines (see lane 6 in Fig. 5B and 5C). In contrast, CAM 6-E7.2βg-8 pre-mRNA gave rise to small but reproducible inclusion of middle exon in MDA-MB468 cell line, but only exon skipped mRNA in ZR75 (see lane 7 in Fig. 5B and 5C). These data imply that exonic sequences in the immediate vicinity of the 5' and 3' ss play an important role in the splicing of exon 7.

### Regulatory elements in exon 7 and flanking intronic sequences are important for controlling CEACAM1 alternative splicing

**Figure 6:**
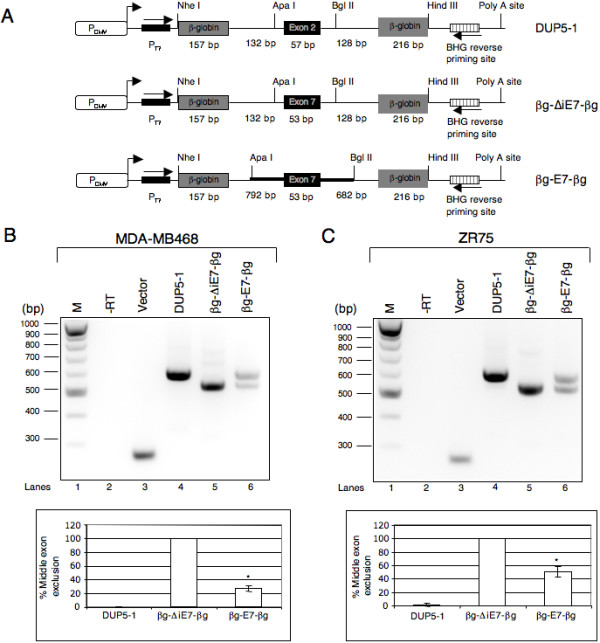
**Regulatory elements in Exon 7 and flanking intronic sequences are important for controlling CEACAM1 alternative splicing.** (A) The DUP5-1 minigene shown here contains three exons and two introns derived from human β-globin gene and is essentially identical to previously described version [46] except that vector backbone is pcDNA 3.1/Myc-His(-) C. The middle exon is a chimera of β-globin exon 1 and 2. The name of each minigene and exon and intron sizes are indicated. Minigene βg-ΔiE7-βg is a derivative of DUP5-1 in which the middle exon 2 is replaced by exon 7 of CEACAM1. Minigene βg-E7-βg was created by digesting pDUP5-1 with ApaI and BglII and inserting PCR amplified CEACAM1 fragment that contains exon 7 and the flanking introns 6 and 7 (thick line) except first 150 bp in intron 6 and last 94 bp in intron 7. (B and C) RT-PCR analysis of MDA-MB468 and ZR75 cells transfected with the indicated minigenes or empty vector. -RT represents omission of reverse transcriptase during cDNA synthesis. The data shown is representative of three independent experiments. Bar diagrams represent the mean ± SD of at least three independent experiments. *P < 0.001 versus DUP5-1.

To further investigate the mechanism of exon 7 splicing regulation we tested the splicing of CEACAM1 exon 7 in a heterologous context in complete absence of flanking exons, but either lacking or containing majority of the flanking intron sequences. To this end, we cloned CEACAM1 exon 7 without (βg-ΔiE7-βg) or with (βg-E7-βg) the flanking intron sequences between two constitutive β-globin exons of the pDUP5-1 (Fig. 6A). Minigene βg-ΔiE7-βg consists entirely of β-globin-derived sequences, with the exception of 53 nucleotides of exon 7 that replaced the central exon. On the other hand, minigene βg-E7-βg contains entire exon 7 and the flanking intron sequences with the exception of 5' and 3' ss of exon 6 and 8, respectively. These reporters were transiently expressed in MDA-MB468 and ZR75 cells and after 24 h total RNA was isolated followed by RT-PCR with vector specific primers. Consistent with a previously published report [46], DUP5-1 generated transcripts gave rise to constitutively spliced mRNA in both cell lines as evident by the formation of ~600 bp product (see lane 4 in Fig. 6B and 6C). The splicing of βg-ΔiE7-βg transcripts also produced a single mRNA species with a molecular size of ~500 bp. Since the expected size of exon 7 included mRNAs is ~600 bp, the observed ~500 bp product is indicative of exon 7 skipping (Fig. 6B and 6C, cf. lane 5). To verify that the splicing of βg-ΔiE7-βg pre-mRNA resulted in exon 7 skipped mRNA, ~500 bp band was excised and subjected to DNA sequencing. Examination of sequencing results (data not shown) confirmed that βg-ΔiE7-βg pre-mRNA gave rise to exon 7-skipped mRNA in both cell lines. Interestingly, in both cell lines the splicing of βg-E7-βg transcripts gave rise to mRNAs with or without exon 7, suggesting that the flanking intron sequences play a vital role in the regulation of CEACAM 1 alternative splicing (see lane 6, Fig. 6B and 6C). To confirm that these mRNAs were not generated due to cryptic splice site activation, the identity of each mRNA was confirmed by DNA sequencing (data not shown). We conclude that sequences residing in exon 7 as well as flanking introns are required for the regulation of CEACAM1 alternative splicing.

## Discussion

CEACAM1 plays an important role in many aspects of epithelial cell function. Although the role of CEACAM1 as a tumor suppressor gene in colon and prostate has been investigated in great detail, its exact role in breast cancer is less well defined. For example, while downregulation of CEACAM1 gene has been suggested to contribute to the development of >90% of colon cancers [43], the lack of CEACAM1 expression account for ~30% of breast cancer [45]. In fact, we have recently shown that forced expression of CEACAM1-S in the mammary epithelial cell line MCF7, which only expresses a small amount of the long splice variant, reverts the growth of these cells to a normal acinar morphology in 3D culture [29]. Thus, it appears that restoration of expression of the short splice variant is important for the normal function of breast epithelial cells. This idea is supported by the analysis of short and long splice variants in normal breast tissue where the ratio of S:L was ~15–16. In the case of breast cancers, the ratio of S:L was between 1.0 to 7.8, but never reached the level found in normal breast (Fig. 4). Thus, the increased expression of the long splice variant may be a prognostic indicator for breast cancer. However, it should be noted that this study was performed at the mRNA level and requires further examination of the protein level, an analysis that requires the use of isoform-specific antibodies.

The importance of the expression of the short cytoplasmic domain splice variant in normal breast requires some thought, since it is the long splice variant that contains two ITIMs which are expected to convey inhibitory functions. Indeed, this is the case for T-cells, where the long isoform is exclusively expressed and its presence inhibits T-cell proliferation and release of IL-2 [47-49]. The absence of inhibitory motifs or association with inhibitory molecules would seem to predict that the short splice variant would not act as a tumor suppressor, but instead, it appears that the opposite is true. For example, CEACAM1 is a negative prognosticator in melanoma [50], and we have found that only the long cytoplasmic domain splice variant is expressed in this cancer (unpublished results). Thus, CEACAM1 splice variants may act in a tissue specific manner where the context of cell signaling may be very different from one cell or tissue to another. Furthermore, the association of the short splice variant with the cytoskeleton may be a key factor, since abnormalities in the cytoskeleton are a common defect in cancers [51].

In consideration of a role for different ratios of the short and long cytoplasmic domain splice variants, a recent study has shown in hepatocytes and polarized kidney cells that the long isoforms are shuttled to cell-cell boundaries and the short isoforms to the luminal surface [52]. Thus, it appears that the two types of isoforms have distinct roles in tissue organization. Indeed, we find that forced expression of the short isoform in MCF7 cells grown in 3D culture leads to expression at the luminal surface [29]. It can be speculated that alteration of physiologically important ratios of S:L isoforms would disrupt epithelial cell polarization, which is a hallmark of epithelial cell cancers [51,53].

Alternative splicing is a widespread phenomenon that expands the size of human proteome and generates proteins with functionally diverse properties. A number of human genes employ alternative splicing to express physiologically important levels of mRNA isoforms that play a crucial role in cell homeostasis. In fact, several recent studies have indicated that a slight change in the delicate balance of mRNA splice variants is associated with human genetic diseases and various forms of cancer (reviewed in Ref [8,16]). However, the underlying mechanisms regulating the alternative splicing of genes associated with human diseases are not fully understood.

Previous studies aimed at understanding the function of human CEACAM1 for most part examined the expression of CEACAM1-L mRNA or protein without taking into account the potential contribution of short cytoplasmic domain splice variant or effect of the ratios between the long and short cytoplasmic domain splice variants. Here in this study, we find that the pattern of CEACAM1 splicing and the ratios of S:L splice variants vary in a variety of normal human tissues. Importantly, compared to normal tissues, alterations in the ratios of CEACAM1 cytoplasmic domain isoforms are observed in cancer cell lines and breast cancer specimens (Fig. 2, 3, 4). In an effort to identify *cis*-acting regulatory elements that control the alternative splicing of CEACAM1, we constructed a series of minigenes and examined their splicing by transiently transfecting two breast cancer cell lines, MDA-MB468 and ZR75. We observed that substitution of β-globin exon 2 sequences for exon 7 (without changing the size of the exon) resulted in the skipping of middle exon (Fig. 5B and 5C, cf lane 5). Likewise, replacement of the first or the last 20 residues in exon 7 by the corresponding β-globin exon 2 sequences failed to prevent exon skipping (Fig. 5B and 5C, see lanes 6–7). These data suggest the presence of regulatory *cis*-acting elements that promote exon 7 splicing. However, skipping of exon 7 in the context of a β-globin reporter construct indicate that additional regulatory elements within the flanking introns contribute to the splicing of exon 7 (see lane 5 in Fig. 6B and 6C). Support to this interpretation comes from the observation that β-globin reporter (βg-E7-βg) comprising of exon 7 and flanking intron sequences recapitulate the alternative splicing of CEACAM1 in both breast cancer cell lines (Fig. 6B and 6C, lane 6). Together these data strongly suggest that a complex regulatory network involving interactions between sequence elements in exon 7 and the flanking introns may be required for the splicing of exon 7. Clearly, a detailed experimental analysis will be required to map the regulatory elements necessary for the splicing of CEACAM1.

## Conclusion

The present study was undertaken to better understand the relationship between CEACAM1 expression and neoplasia. Our results show that CEACAM1 is expressed in a tissue dependent manner with significant differences in the ratios of its short (CEACAM1-S) and long (CEACAM1-L) cytoplasmic domain splice variants. Our data in human breast cancer show a higher ratio of S:L in normal versus malignant tissues, raising the possibility that tumor suppressive role of CEACAM1 may actually depend on which isoform is dominant. In addition, we have identified two *cis*-acting elements required for the regulation of CEACAM1 splicing into long or the short cytoplasmic domain isoforms. These sequences could serve as a target to modulate CEACAM1 alternative splicing, which may have important therapeutic implications.

## Methods

### Plasmid construction

All DNA constructs were generated by standard cloning procedures and confirmed by sequencing. The minigene-splicing cassette CAM 6-7-8 was constructed by amplifying human genomic sequences from exon 6 to exon 8 with Pfu DNA polymerase (Stratagene) and primers #40563 (5'-AAAAAAAAGCTAGCCTCGAGATAATGCTCTACCACAAGAAAATGG-3') and #41658 (5'-AACTGCAGAAGCTTCCTTGTTAGGTG-3'). The underline sequences represent NheI and HindIII restriction enzyme sites. The PCR amplified DNA was digested with NheI and HindIII (New England Biolabs) and cloned into pcDNA 3.1/Myc-His(-) C (Invitrogen). The CAM 6-βg-8 minigene was engineered by two-step overlapping PCR approach using CAM 6-7-8 minigene as template. In the first step, the upstream and the downstream portions of the minigene were generated by using primers # 40563 and #45361 (5'-AAAGAACCTCTGGGTCCAAGGGTAGACCACCAGCAGCCTAAATAAATATCAGAAACTGTG-3') and primers #41658 and #45360 (5'-TTGGACCCAGAGGTTCTTTGAGTCCTTTGGGGATCGTAAGTAAAGCCACTTACCC-3'), respectively. The first step PCR products were used as template for the overlapping PCR with primers #40563 and #41658. The amplified fragment was digested with NheI and HindIII, gel purified, and ligated to pcDNA 3.1/Myc-His(-) C linearized with appropriate restriction enzymes. The minigenes CAM 6-E7.1βg-8 and CAM 6-E7.2βg-8 in which the first and the last 20 nt of exon 7 were replaced by β-globin sequences were essentially made following two-step overlapping PCR-based cloning using pCAM 6-7-8 as a template. The following primer sets were used to generate indicated constructs:

CAM 6-E7.1βg-8: #46888-ET7 (5'-GAAATTAATACGACTCACTATAGGG-3'); #48125 (5'-AAGGGTAGACCACCAGCAGCCTAAATAAATATCAGAAACTGTG-3'); #48124 (5'-GCTGCTGGTGGTCTACCCTTTCACAGAGCACAAACCCTCAG-3'); #46889-EBGH-R (5'-AACTAGAAGGCACAGTCGAGGC-3').

CAM 6-E7.2βg-8: #46888-ET7; #48127 (5'-GATCCCCAAAGGACTCAAAGTTGTGCTCTGTGAGATCACG-3'); #48126 (5'-CTTTGAGTCCTTTGGGGATCGTAAGTAAAGCCACTTACCCC-3'); #46889-EBGH-R. The minigene DUP5-1 has been previously described [46] except that the sequence from 549 to 1228 was PCR amplified using primers #47240 (5'-AAACCGCTCGAGGCTAGCTCAGCTTGCTTACATTTGCTTCTG-3') and #47241 (5'-AACTGCAGAAGCTTGGATCCACGTGCAGCTTGTCAC-3'), and NheI/HindIII digested fragment cloned in pcDNA 3.1/Myc-His(-) C. The minigenes βg-ΔiE7-βg and βg-E7-βg are derivatives of pDUP5-1. The plasmid pβg-ΔiE7-βg was constructed by overlapping PCR using pDUP5-1 as template and primers #47240; #48386 (5'-TCTGTGAGATCACGCTGGTCGCTTGCCCTAAGGGTGGGAAAATAGAC-3'); #47241 and #48387 (5'-GACCAGCGTGATCTCACAGAGCACAAACCCTCAGTCTCCAACCACAGTTGGTATCAAGGTTACAAGA-3'). The NheI/HindIII digested fragment was cloned in pcDNA 3.1/Myc-His(-) C. Plasmid pβg-E7-βg was created by digesting pDUP5-1 with ApaI and BglII and inserting CEACAM1 fragment from 151 to 1500 (1 represents the first nucleotide in intron 6), which was amplified by PCR using pDUP5-1 as template and primers #43355 (5'-AGCT GGGCCCGTTCTGTTTCCTCCAAGGC-3') and #43356 (5'-GGATAGATCTAGCTGAAGGAGGTGGC-3'). The underline sequences in #43355 and #43356 represent recognition sequence for ApaI and BglII restriction enzymes.

### Cell culture

The human breast (BT474, MCF7, MDA-MB468 and ZR75) and prostate (PC3, LNCaP and DU-145) cancer cell lines were obtained from American Type Culture Collection (Manassas, VA). The glioma cell lines U251T, T98G and U87MG and colon cancer cell lines HT29, HCT116 (-/+ p53), S1 and HCC2998 were kind gifts from Drs. John Shively and Y. Yen (City of Hope). The breast cancer cell lines, with the exception of MDA-MB468, are ER positive, express HER2/neu, and were derived from metastatic sites. The prostate cancer cell lines used in this study are metastatic. While LNCaP is hormone sensitive, PC3 and DU-145 lines are hormone-refractory. The human glioma cell lines U251T is the tumorigenic strain of U251. The line U87MG is derived from malignant gliomas and unlike T98G it is considered to be tumorigeneic. The human colon cancer cell lines HT29, HCT116 and HCC2998 are tumorigenic. The S1 is a human colon cancer cell line derived from the S1 clone of tumorigenic LS-180 colon carcinoma cell line. These cell lines were culture in ATCC recommended cultured conditions using following growth mediums (RPMI 1640, MEM containing 2% glutamine, DMEM with high glucose or McCoy's medium) supplemented with 10% heat-inactivated fetal bovine serum (Omega Scientific Inc., CA) at 37°C in humidified air containing 5% CO_2_.

### Transient transfection, RNA isolation and RT-PCR

A day prior to transfection, 5 × 10^5 ^MDA-MB468 or ZR75 cells were seeded on a 6 well plate. Next day, 2.5 μg plasmid was transfected with Lipofectamine 2000 (Invitrogen) following manufacturer's instructions. After 24 hours, cells were harvested and total RNA was isolated using RNeasy total RNA isolation kit (Qiagen) and treated with DNase I (Roche). Total RNA (3–5 μg) was subjected to reverse transcription with oligo (dT)_20 _and MMLV reverse transcriptase (Invitrogen) essentially according to the manufacturer's instructions. Next, 1 μL of RT mixture was PCR amplified using vector specific primers ET7 (5'-GAAATTAATACGACTCACTATAGGG-3') and EBGH-R (5'-AACTAGAAGGCACAGTCGAGGC-3'). The PCR amplification conditions were as follows: 94°C for 5 min; 30 cycles at 94°C for 45 sec, 60°C for 1 min, and 72°C for 1 min; and a final extension at 72°C for 10 min. The PCR amplified products were analyzed by electrophoresis on a 2.5% agarose gel, and quantified by ImageJ software version 1.36b (National Institutes of Health).

### Analysis of CEACAM1 splicing in normal human tissues, breast cancer specimens and cancer cell lines

To examine the expression of endogenous CEACAM1 long and short cytoplasmic domain mRNAs, we isolated total RNA from breast cancer specimens and the non-diseased breast tissues that were deposited in City of Hope tissue bank. The breast tumors of all of the treated patients were removed by complete surgical resection at City of Hope and a written consent was received from patients or family members. The institutional review board approved the research protocol. The clinical history of breast cancer specimens is detailed below. The specimen no. 1, 3, 5, 6 and 9 are invasive ductal adenocarcinoma; 2 and 8 ductal carcinoma; and remainder designated as breast carcinoma. All of the ten patients showed lymph node metastases of breast cancer and tumors were moderate-to-poorly differentiated. Typically ~30 mg frozen tissue sample or 5 × 10^5 ^pelleted cells were homogenized followed by the isolation of total RNA using RNeasy kit essentially as described in the protocol (Qiagen). Total RNA from normal human tissues (brain, colon, heart, kidney, liver, lung and prostate) were obtained from Ambion (Austin, TX) and directly used for reverse-transcription-PCR as described above, except that oligonucleotide # 43309 (5'-GGTTGCTCTGATAGCAGTAG-3') and # 43310 (5'-AGCCTGGAGATGCCTATTAG-3') were used as forward and reverse primers, respectively. Since the binding sites for forward and reverse primers are located in exon 6 and the 3' UTR, respectively, which are interrupted by three introns, the presence of contaminating genomic DNA amplification would be easily detected. In addition, we followed previously reported PCR amplification conditions that represent linear amplification of both mRNA species in a single reaction [39].

### Statistical Analysis

The results obtained in this study represent mean ± SD from at least three independent experiments. The statistical significance of data groups was performed with Student's *t*-test.

## Competing interests

The authors declare that they have no competing interests.

## Authors' contributions

RKG performed designing and construction of CEACAM1 mingenes, provided overall direction to the project and prepared manuscript, SG performed transient transfection experiments and statistical analysis, JES helped in experimental advice and manuscript preparation, YY provided cancer tissue specimens and critical reading of the manuscript. The manuscript has been read and approved by all authors.
